# Supplementary material for: Modeling glioblastoma heterogeneity as a dynamic network of cell states
Source: Mol Syst Biol. 2021 Sep 16;17(9):e10105. doi: 10.15252/msb.202010105 (PMC8444284; doi:10.15252/msb.202010105)
Supplement: Supplementary file 6 — Source Data for Figure 5 [file MSB-17-e10105-s004.zip › Figure5A_sourcedata/GSEA_3071/nosigdb_stateIII.GseaPreranked.1622795155562/DOROSZKO_U3180MG_CLUSTER_3.html]

Details for gene set DOROSZKO\_U3180MG\_CLUSTER\_3[GSEA]

|  || Dataset | state33071 |
| Phenotype | NoPhenotypeAvailable |
| Upregulated in class | na\_pos |
| GeneSet | DOROSZKO\_U3180MG\_CLUSTER\_3 |
| Enrichment Score (ES) | 0.17919599 |
| Normalized Enrichment Score (NES) | 2.5110242 |
| Nominal p-value | 0.0 |
| FDR q-value | 5.6552707E-4 |
| FWER p-Value | 0.005 |
Table: GSEA Results Summary

  

Fig 1: Enrichment plot: DOROSZKO\_U3180MG\_CLUSTER\_3      
 Profile of the Running ES Score & Positions of GeneSet Members on the Rank Ordered List

  

| PROBE | GENE SYMBOL | GENE\_TITLE | RANK IN GENE LIST | RANK METRIC SCORE | RUNNING ES | CORE ENRICHMENT || 1 | EEF1B2 |  |  | 28 | 0.501 | 0.0038 | Yes |
| 2 | NDUFA6 |  |  | 54 | 0.458 | 0.0074 | Yes |
| 3 | PDCD5 |  |  | 77 | 0.435 | 0.0114 | Yes |
| 4 | SNRPB |  |  | 122 | 0.395 | 0.0086 | Yes |
| 5 | COX5A |  |  | 131 | 0.389 | 0.0152 | Yes |
| 6 | SMS |  |  | 149 | 0.375 | 0.0191 | Yes |
| 7 | MRPL17 |  |  | 159 | 0.368 | 0.0249 | Yes |
| 8 | SNRPF |  |  | 160 | 0.367 | 0.0331 | Yes |
| 9 | GLO1 |  |  | 161 | 0.367 | 0.0412 | Yes |
| 10 | PSMC3 |  |  | 162 | 0.366 | 0.0494 | Yes |
| 11 | CACYBP |  |  | 165 | 0.364 | 0.0570 | Yes |
| 12 | NME1 |  |  | 166 | 0.364 | 0.0651 | Yes |
| 13 | MZT2A |  |  | 187 | 0.348 | 0.0676 | Yes |
| 14 | ATP5MC1 |  |  | 208 | 0.334 | 0.0698 | Yes |
| 15 | SUMO3 |  |  | 210 | 0.333 | 0.0769 | Yes |
| 16 | RANBP1 |  |  | 221 | 0.328 | 0.0816 | Yes |
| 17 | NUDC |  |  | 226 | 0.325 | 0.0878 | Yes |
| 18 | UQCC2 |  |  | 234 | 0.320 | 0.0931 | Yes |
| 19 | MZT2B |  |  | 244 | 0.313 | 0.0977 | Yes |
| 20 | LSM4 |  |  | 268 | 0.303 | 0.0984 | Yes |
| 21 | GTF3A |  |  | 299 | 0.290 | 0.0970 | Yes |
| 22 | POLE3 |  |  | 301 | 0.290 | 0.1032 | Yes |
| 23 | TIMM10 |  |  | 304 | 0.290 | 0.1091 | Yes |
| 24 | CMSS1 |  |  | 307 | 0.289 | 0.1150 | Yes |
| 25 | PA2G4 |  |  | 308 | 0.289 | 0.1215 | Yes |
| 26 | POLR2I |  |  | 311 | 0.289 | 0.1274 | Yes |
| 27 | EIF4EBP1 |  |  | 316 | 0.287 | 0.1327 | Yes |
| 28 | BANF1 |  |  | 317 | 0.287 | 0.1391 | Yes |
| 29 | MINOS1 |  |  | 321 | 0.286 | 0.1447 | Yes |
| 30 | CSNK2B |  |  | 333 | 0.283 | 0.1481 | Yes |
| 31 | CLIC1 |  |  | 373 | 0.269 | 0.1439 | Yes |
| 32 | SNRPD1 |  |  | 376 | 0.268 | 0.1493 | Yes |
| 33 | H2AFZ |  |  | 386 | 0.265 | 0.1529 | Yes |
| 34 | FKBP3 |  |  | 387 | 0.264 | 0.1587 | Yes |
| 35 | AP2S1 |  |  | 398 | 0.260 | 0.1619 | Yes |
| 36 | TPRKB |  |  | 399 | 0.260 | 0.1677 | Yes |
| 37 | VPS29 |  |  | 402 | 0.259 | 0.1729 | Yes |
| 38 | CHTF18 |  |  | 444 | -0.251 | 0.1678 | Yes |
| 39 | MTFR2 |  |  | 461 | -0.252 | 0.1692 | Yes |
| 40 | MNS1 |  |  | 463 | -0.252 | 0.1745 | Yes |
| 41 | RAD54L |  |  | 497 | -0.254 | 0.1715 | Yes |
| 42 | CDC45 |  |  | 506 | -0.255 | 0.1751 | Yes |
| 43 | HJURP |  |  | 513 | -0.255 | 0.1792 | Yes |
| 44 | OIP5 |  |  | 537 | -0.256 | 0.1789 | No |
| 45 | NDC80 |  |  | 636 | -0.261 | 0.1589 | No |
| 46 | MCM4 |  |  | 638 | -0.261 | 0.1645 | No |
| 47 | SGO1 |  |  | 674 | -0.263 | 0.1612 | No |
| 48 | FUCA2 |  |  | 884 | -0.274 | 0.1124 | No |
| 49 | C21orf58 |  |  | 946 | -0.276 | 0.1025 | No |
| 50 | DHFR |  |  | 956 | -0.277 | 0.1063 | No |
| 51 | HELLS |  |  | 970 | -0.278 | 0.1091 | No |
| 52 | MCM3 |  |  | 1021 | -0.280 | 0.1022 | No |
| 53 | CDCA3 |  |  | 1029 | -0.281 | 0.1066 | No |
| 54 | FOXM1 |  |  | 1102 | -0.285 | 0.0941 | No |
| 55 | PCNA |  |  | 1116 | -0.286 | 0.0970 | No |
| 56 | RMI2 |  |  | 1249 | -0.292 | 0.0689 | No |
| 57 | NUSAP1 |  |  | 1262 | -0.293 | 0.0722 | No |
| 58 | DTL |  |  | 1297 | -0.295 | 0.0699 | No |
| 59 | KNL1 |  |  | 1355 | -0.298 | 0.0615 | No |
| 60 | XRCC2 |  |  | 1435 | -0.302 | 0.0475 | No |
| 61 | GGH |  |  | 1439 | -0.302 | 0.0535 | No |
| 62 | MMS22L |  |  | 1494 | -0.306 | 0.0461 | No |
| 63 | KIF4A |  |  | 1504 | -0.306 | 0.0506 | No |
| 64 | NR2C2AP |  |  | 1526 | -0.308 | 0.0519 | No |
| 65 | EXO1 |  |  | 1537 | -0.308 | 0.0561 | No |
| 66 | MACROD1 |  |  | 1540 | -0.308 | 0.0625 | No |
| 67 | DEPDC1 |  |  | 1564 | -0.310 | 0.0633 | No |
| 68 | MYBL2 |  |  | 1587 | -0.311 | 0.0645 | No |
| 69 | PKMYT1 |  |  | 1591 | -0.311 | 0.0706 | No |
| 70 | SGO2 |  |  | 1659 | -0.316 | 0.0601 | No |
| 71 | GNG4 |  |  | 1706 | -0.319 | 0.0551 | No |
| 72 | HAUS8 |  |  | 1741 | -0.321 | 0.0533 | No |
| 73 | NUF2 |  |  | 1755 | -0.322 | 0.0571 | No |
| 74 | WDR76 |  |  | 1795 | -0.324 | 0.0541 | No |
| 75 | BRCA1 |  |  | 1844 | -0.327 | 0.0487 | No |
| 76 | MCUB |  |  | 1884 | -0.329 | 0.0458 | No |
| 77 | UBE2C |  |  | 1942 | -0.332 | 0.0382 | No |
| 78 | SMC2 |  |  | 1963 | -0.333 | 0.0404 | No |
| 79 | RRM2 |  |  | 2147 | -0.345 | 0.0000 | No |
| 80 | PAQR4 |  |  | 2150 | -0.345 | 0.0072 | No |
| 81 | BRCA2 |  |  | 2213 | -0.350 | -0.0013 | No |
| 82 | TPX2 |  |  | 2289 | -0.356 | -0.0131 | No |
| 83 | ZNF367 |  |  | 2292 | -0.356 | -0.0056 | No |
| 84 | MCM2 |  |  | 2303 | -0.357 | -0.0003 | No |
| 85 | ASF1B |  |  | 2312 | -0.358 | 0.0056 | No |
| 86 | DEPDC1B |  |  | 2354 | -0.362 | 0.0029 | No |
| 87 | CDC7 |  |  | 2539 | -0.376 | -0.0371 | No |
| 88 | TACC3 |  |  | 2560 | -0.379 | -0.0339 | No |
| 89 | FAM111B |  |  | 2585 | -0.381 | -0.0317 | No |
| 90 | FADS1 |  |  | 2588 | -0.381 | -0.0238 | No |
| 91 | CCNA2 |  |  | 2635 | -0.385 | -0.0273 | No |
| 92 | EMP2 |  |  | 2695 | -0.390 | -0.0341 | No |
| 93 | CDCA7 |  |  | 2710 | -0.391 | -0.0290 | No |
| 94 | E2F8 |  |  | 2724 | -0.393 | -0.0237 | No |
| 95 | FAM111A |  |  | 2761 | -0.396 | -0.0244 | No |
| 96 | ABHD3 |  |  | 2805 | -0.400 | -0.0267 | No |
| 97 | DDB2 |  |  | 2834 | -0.402 | -0.0251 | No |
| 98 | SMC4 |  |  | 2872 | -0.405 | -0.0258 | No |
| 99 | MXD3 |  |  | 2881 | -0.406 | -0.0189 | No |
| 100 | CSRP2 |  |  | 2974 | -0.417 | -0.0338 | No |
| 101 | FANCA |  |  | 2976 | -0.417 | -0.0247 | No |
| 102 | ASRGL1 |  |  | 2986 | -0.418 | -0.0178 | No |
| 103 | INAFM1 |  |  | 2988 | -0.418 | -0.0087 | No |
| 104 | TEDC1 |  |  | 3030 | -0.424 | -0.0101 | No |
| 105 | FANCD2 |  |  | 3065 | -0.427 | -0.0095 | No |
| 106 | ATAD2 |  |  | 3115 | -0.433 | -0.0127 | No |
| 107 | MCM8 |  |  | 3129 | -0.435 | -0.0064 | No |
| 108 | CCNE2 |  |  | 3138 | -0.436 | 0.0012 | No |
| 109 | CDCA2 |  |  | 3212 | -0.444 | -0.0081 | No |
| 110 | SLC20A1 |  |  | 3344 | -0.470 | -0.0320 | No |
| 111 | METRN |  |  | 3358 | -0.472 | -0.0249 | No |
| 112 | BRIP1 |  |  | 3380 | -0.475 | -0.0199 | No |
| 113 | OLFM1 |  |  | 3398 | -0.480 | -0.0136 | No |
| 114 | GTSE1 |  |  | 3468 | -0.498 | -0.0207 | No |
| 115 | NCAPG |  |  | 3513 | -0.512 | -0.0208 | No |
| 116 | CALU |  |  | 3566 | -0.533 | -0.0226 | No |
| 117 | HIST1H1D |  |  | 3637 | -0.569 | -0.0283 | No |
| 118 | TOP2A |  |  | 3671 | -0.594 | -0.0237 | No |
| 119 | MKI67 |  |  | 3788 | -0.721 | -0.0381 | No |
| 120 | CENPF |  |  | 3842 | -0.820 | -0.0338 | No |
| 121 | ASPM |  |  | 3899 | -1.166 | -0.0225 | No |
| 122 | HIST1H4C |  |  | 3914 | -1.352 | 0.0039 | No |
Table: GSEA details [plain text format]

  

Fig 2: DOROSZKO\_U3180MG\_CLUSTER\_3: Random ES distribution      
 Gene set null distribution of ES for **DOROSZKO\_U3180MG\_CLUSTER\_3**
